# Pericarditis in Infants

**Published:** 1912-12

**Authors:** 


PERICARDITIS IN INFANTS. Nobecourt, *Gaz. des Pract.*, Sept. 1, 1912. In nurslings and children up to the age of six, acute purulent cases are especially frequent, tributary to non-specific, pneumococcic, streptococcic and staphylococcic infections. They frequently follow pneumonia, broncho-pneumonia, measles, scarlet fever, whooping cough, etc. Rheumatic and tuberculous forms are rare. Acute rheumatic infection is frequent after the age of six or seven and is usually dry. In about half the cases, both pericardium and endocardium are involved. Rheumatic purulent pericarditis never occurs.

